# DIFFERENCES IN ADVANCE CARE PLANNING AMONG RACIAL AND ETHNIC MINORITIES AND PERSONS WITH ADRD

**DOI:** 10.1093/geroni/igac059.1479

**Published:** 2022-12-20

**Authors:** Jessica Yauk, Hongdao Meng, Debra Dobbs

**Affiliations:** University of South Florida, Tampa, Florida, United States; University of South Florida, Tampa, Florida, United States; University of South Florida, Tampa, Florida, United States

## Abstract

Advance care planning (ACP) is an important process to enable individuals to make their preferences known in the event of dementia onset. Racial and ethnic minorities have an increased risk of developing dementia. Understanding more about ACP differences by race and dementia status will help improve ACP communication among minority populations. For the current study the 2018 wave of the Health and Retirement Study (N=8,663) to test the relationship between dementia status and ACP and if there were differences by race. Findings indicated that both African Americans and Hispanics are less likely to have completed ACP than non-Hispanic Whites. In unadjusted models, having a dementia diagnosis indicated a lower likelihood of completing ACP; dementia was not significant in the final model when controlling for predisposing and enabling factors. More research on the predisposing and enabling factors that influence ACP among minority and dementia populations is needed.

